# Preoperative imaging optimized for epiretinal membrane surgery

**DOI:** 10.1186/s40942-021-00304-w

**Published:** 2021-04-13

**Authors:** Elise Philippakis, Raphaël Thouvenin, Sarra Gattoussi, Aude Couturier, Ramin Tadayoni

**Affiliations:** Ophthalmology Department, AP-HP, Hôpital Lariboisière, Université de Paris, 75010 Paris, France

**Keywords:** Epiretinal membrane, En face OCT, Blue staining, Blue reflectance, Intraoperative visualization

## Abstract

**Background:**

To compare imaging modalities for visualizing primary epiretinal membrane (ERM) with each other and with intraoperative digital images (IDI) after blue staining.

**Methods:**

The records of consecutive patients operated for primary ERM over a 12-month period were retrospectively reviewed. Preoperative imaging included color fundus photography (CFP), En Face spectral-domain optical coherence tomography (OCT), 45° infrared- (IR) and blue-reflectance (BR) scanning laser ophthalmoscopy. All images were qualitatively analyzed and scored from 0–4 according to the ability to visualize ERM details (0 = no visible ERM or vessel contraction, 1 = vessel contraction, 2 = retinal folds, 3 = ERM limits, 4 = elevated ERM edge). The preoperative ERM morphology was then compared to that seen on the IDI acquired after 1-min blue dye staining when available.

**Results:**

Seventy eyes were included. The highest score for ERM visualization was obtained on BR and En Face OCT. A score of 3 or 4 was obtained in 68.5%, 62.1%, 17.9% and 13.6% of cases on En Face OCT, BR, CFP and IR images, respectively. IDI were available for 20 eyes, and showed a similar ERM morphology compared to preoperative images in most cases: a negative staining pattern corresponded to a plaque on En face OCT in 91% of eyes. However, IDI failed to show the ERM edges in 37.5% of cases.

**Conclusion:**

ERM morphology was better visualized preoperatively by BR and En Face OCT, in a similar way to the IDI after staining. Future intraoperative visualization systems could integrate both imaging modalities overlaid with the IDI for guiding ERM removal instead of staining.

## Introduction

Epiretinal membrane (ERM) is the most common indication for surgery in macular diseases in the elderly with a prevalence ranging between 10 and 30% [1]. The success of the surgical procedure relies on the complete peeling of this thin, often almost transparent, tissue from the retinal surface. Nowadays, most surgeons use visualization agents to better identify ERM limits and to facilitate its complete removal [2]. Preoperative multimodal imaging of ERM also aims at analyzing ERM morphology and limits in order to improve surgical procedure planification and the completeness of ERM peeling. It usually includes color fundus photography (CFP) and an optical coherence tomography (OCT) B-scan. On CFP, the ERM is identified by its reflectivity and the alteration of the macular morphology with retinal folds and vessel constriction. The OCT B-scan shows a taut hyperreflective structure on the inner retinal surface with a macular thickening and retinal folds [3]. Over the last decades, other imaging modalities have been developed and may help to better characterize ERM morphology, based on its reflectivity on blue-reflectance (BR) scanning laser ophthalmoscopy (SLO) or retinal folding on En Face OCT [4, 5]. Thus, the best preoperative imaging technique to be used for ERM visualization and surgery planification should be further evaluated. Live intraoperative visualization after staining is probably considered the best way to analyze ERM morphology. A capture of the ERM visualized after staining provides an intraoperative digital image (IDI) that is comparable to preoperative images. The new intraoperative visualization systems provide images that might be used together with preoperative multimodal images for performing a secured imaging-guided surgical procedure. In this perspective, it is necessary to identify which digital image offers the best preoperative visualization of the ERM.

The aim of this study was first to determine which preoperative image allowed best visualizing the ERM, analyzing its morphology and limits and then to compare it to the ERM morphology visualized on the IDI after blue staining.

## Materials and methods

The study conduct met the tenets of the Declaration of Helsinki. The Ethics Committee of the French Society of Ophthalmology (IRB 00008855 Société Française d’Ophtalmologie IRB#1) approved this retrospective review of patient records. Patient consent was obtained.

The records of all consecutive patients who underwent ERM surgery over a 12-month period in the Ophthalmology department of Lariboisière hospital were retrospectively reviewed.

### Patients

Inclusion criteria were having idiopathic ERM and having undergone complete preoperative multimodal imaging and, when available, intraoperative captures of the macular area before and after staining. Patients operated for secondary ERM (myopic, diabetic, age-related macular degeneration, vitreomacular traction, lamellar hole) were excluded.

### Image acquisition

Preoperative imaging included CFP, 45° infrared-reflectance (IR) and BR SLO images acquired with the Spectralis HRA (Heidelberg Engineering GmbH, Heidelberg, Germany) and OCT images acquired with the Cirrus HD-OCT 5000 (Carl Zeiss Meditec, Inc, Humphrey Division, Dublin, California, USA) with En Face analysis. OCT images included a macular cube of 512 (A-scans) × 128 (B-scans), (20 × 20°, 6 × 6 mm, spacing of 47 µm), 2 high-definition 5-line rasters (30° width horizontally and vertically, 9 mm, spacing of 75 µm). The En face analysis was performed using the Advanced Visualization ILM segmentation (20-µm thick slab) positioned on the retinal surface with contrast adjustment to obtain the best defined En Face image of the retinal folding and ERM limits.

### Preoperative image analysis

**Fig. 1:**
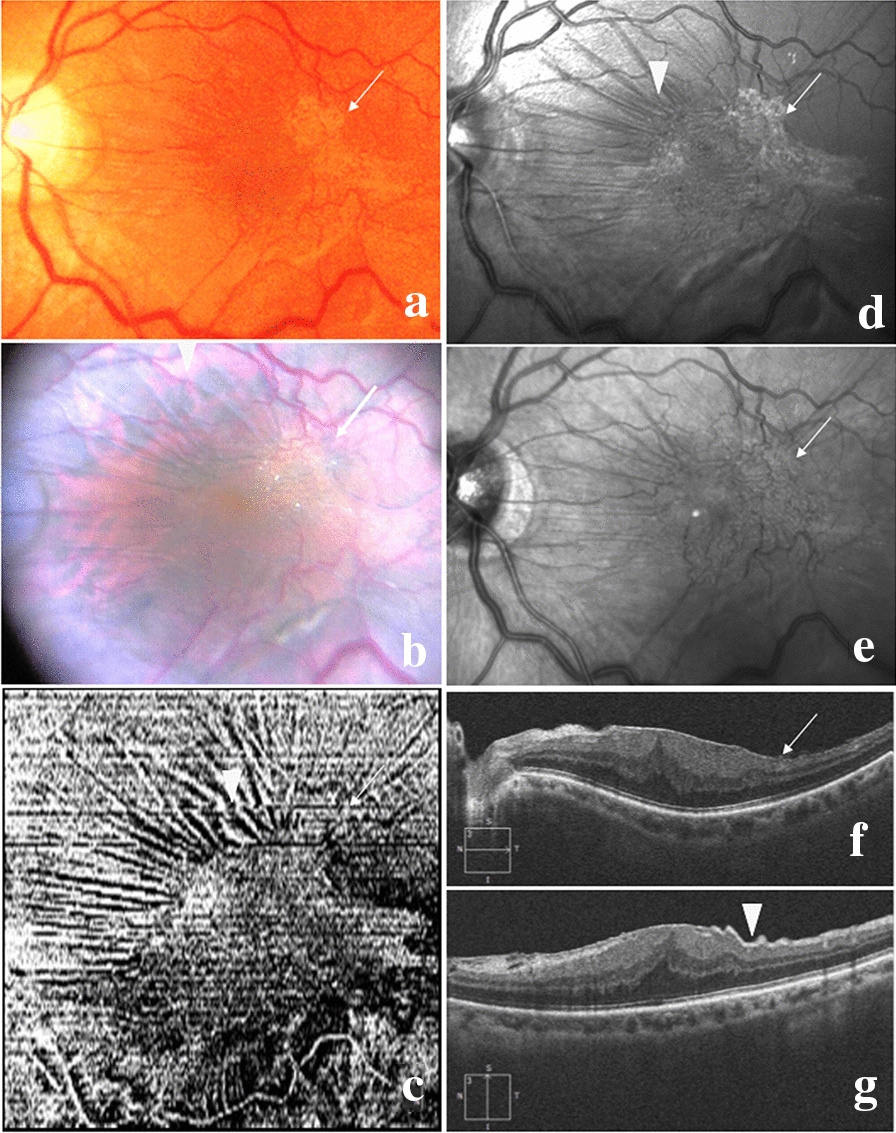
Preoperative and intraoperative images of a case of idiopathic epiretinal membrane (ERM). Color Fundus Photography (**a**) shows the reflectivity of the ERM in the temporo-macular area (arrow). Intraoperatively (**b**), the differential staining of the ILM and ERM allows visualizing ERM limits; the blue dye stains the ILM (arrowhead) but not the ERM, giving this ‘negative’ aspect. En Face OCT (**c**) and Blue Reflectance (**d**) images show a remarkable plaque-like aspect (arrow) surrounded by a retinal folding (arrowhead). The infrared reflectance image (**e**) also shows the ERM (arrow) but its limits are barely visible. Horizontal (**f**) and vertical (**g**) OCT B-scan images show the ERM (arrow), retinal folds (arrowhead) and convex macular thickening

The main outcome was to assess which image provided the best visualization and analysis of ERM morphology and limits. ERM morphology included its contractility, its plaque-like aspect, its edges and the retinal folds. For each patient, 4 images were thus obtained (Fig. 1a, c–e). To be able to compare these multimodal images, each image had to be edited (well rotated and resized) to fit the 45° IR and BR images. Since none of the images could be considered as a gold standard, all images were presented simultaneously to two independent retina specialists (EP, RT). For all cases, each preoperative image was scored by two independent retina specialists (EP, RT) from 0 to 4 according to the quality of ERM visualization: a score of 0 was given when no ERM and vessel contraction were visible; a score of 1 was given when only vessel contraction was observed; a score of 2 was given when the ERM was visible with its retinal folds; a score of 3 was given when the ERM limits were visible and a score of 4 when a clear elevated ERM edge was identified. For each image, the total score was the addition of the scores given by the two retina specialists. The image on which the ERM was considered to be the best visualized was that with the highest score.

### Surgical procedure

The surgical procedure consisted in transconjunctival 25-Gauge 3-port pars plana vitrectomy. After performing core vitrectomy, the macular area was stained with a mix of 0.15% trypan blue and 0.025% brilliant blue G (Membrane-Blue Dual®, DORC, Zuidland, Netherlands) under Balanced Saline Solution for 1 min. In some cases, the best intraoperative visualization of the ERM through a posterior contact lens after staining was acquired (Fig. 1e). The ERM was removed using a 25-gauge + Grieshaber endgrasping forceps (Alcon Grieshaber AG, Scaffhausen, Switzerland). If the ILM was not peeled off with the ERM, it was either actively peeled off or not at surgeon’s discretion. The operating microscopes used were the Zeiss OPMI Lumera 700 and Rescan 700 devices (Zeiss Ltd., Jena, Germany).

### Intraoperative image analysis

Intraoperative image analysis consisted in characterizing the aspect of the ERM on the IDI after a 1-min staining with Membrane blue Dual® and comparing it to the preoperative image allowing the best visualization of the ERM, especially its limits and edges. The aspect of the ERM staining on the IDI was compared to the morphological features of the ERM on preoperative images and qualitatively analyzed by two independent retina specialists (EP, AC) by focusing on the visualization of the ERM limits and edges (Fig. 1).

### Statistical analysis

Statistical analyzes were performed using JMP® pro 12 software. Quantitative variables are presented as a mean ± standard deviation (range). Qualitative variables are presented as a number and ratio. Comparisons between groups were performed using a chi-2 test.

## Results

During the 12-month period, 402 patients underwent ERM surgery. Among them, 278 patients had idiopathic ERM and 124 were excluded for myopic ERM (n = 32), diabetic ERM (n = 54), other secondary ERM (n = 27), lamellar hole (n = 10), and juvenile ERM (n = 1). Among the patients with idiopathic ERM, complete preoperative imaging was available in 70 patients who were included for analysis.

Patient and ERM characteristics are shown in Table 1. Briefly, patient mean age at the time of surgery was 69.2 ± 8.1 years (41–87 years), 69% and 31% underwent vitrectomy and phacovitrectomy (when lens opacification was significant), respectively. Retinal cysts and a pseudohole were present in 13% (9/70) and 7% (5/70) of cases, respectively.

**Table 1:** Pre- and intraoperative characteristics of the 70 patients with primary ERM and complete preoperative imaging

Number of eyes, n	70
Age, years	69.2 ± 8.1 (41–87)
Male gender, n (M/F ratio)	38 (1.2)
Side: right eye, n (%)	37 (53)
Preoperative BCVA, LogMAR Snellen equivalent	0.38 ± 0.19 (0.1–1) 20/50
Lens status, n (%)	
Phakic	49 (70)
Preoperative parameters on the OCT B-scan	
CMT (µm)	451 ± 91 (265–708)
Cysts, n (%)	9 (12.8)
Pseudo-hole, n (%)	5 (7.1)
ERM edge, n (%)	7 (10)
Intraoperative parameters	
Complete PVD, n (%)	59 (84)
ILM peeling, n (%)	52 (74.3)

Quantitative values are presented as a mean ± standard deviation (range)

*BCVA* best-corrected visual acuity, *CMT* central macular thickness, *ERM* epiretinal membrane, *ILM* internal limiting membrane, *PVD* posterior vitreous detachment

### Preoperative image analysis (Fig. 2)

**Fig. 2:**
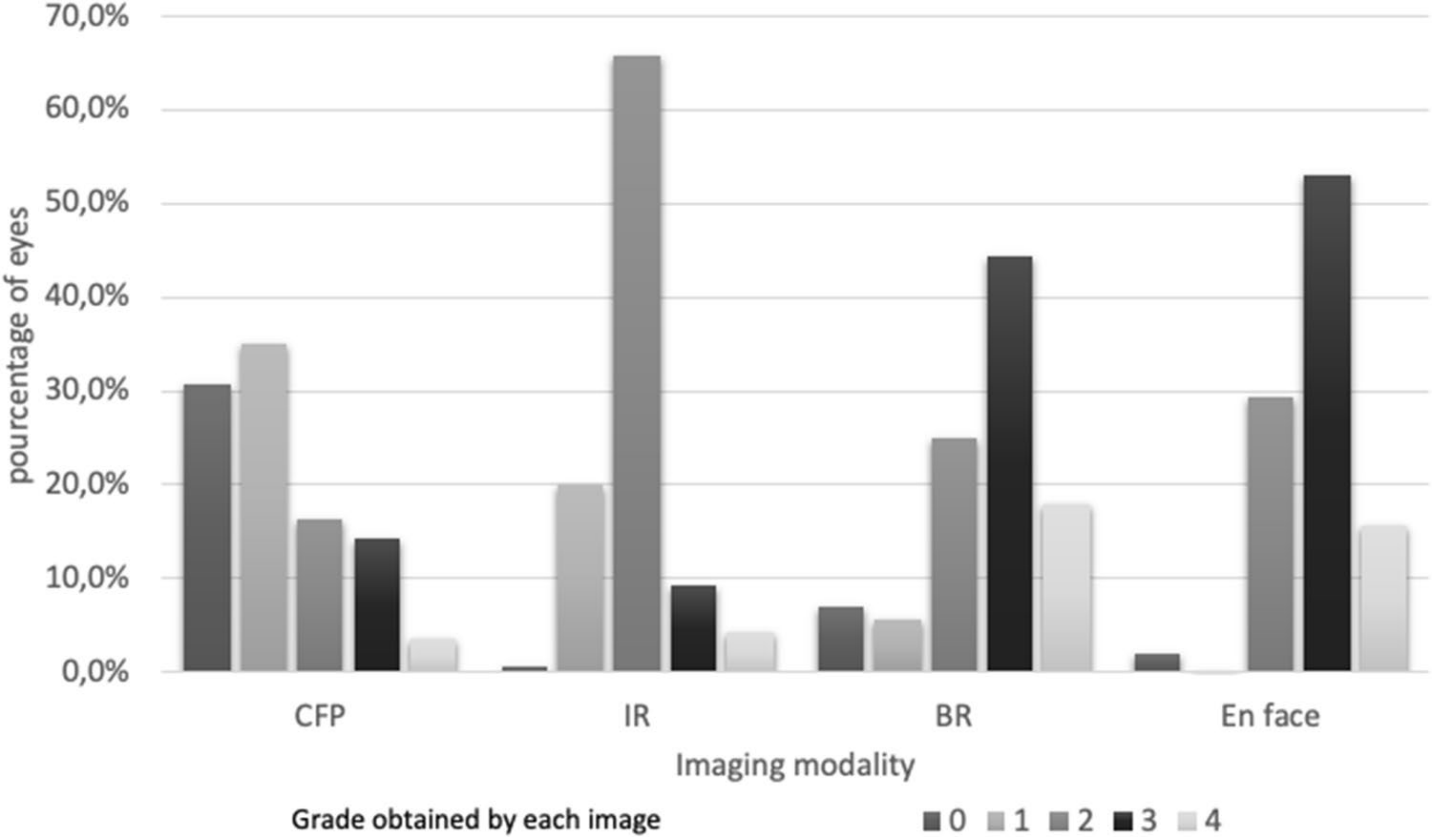
Distribution of the scores for ERM visualization for each type of preoperative image. In 70 eyes, each preoperative image was scored from 0 to 4 according to the quality of ERM visualization: 0 = ERM not visible, 1 = vessel contraction, 2 = retinal folds, 3 = ERM limits, 4 = elevated ERM edge. Color fundus photography (CFP) obtained the score of 0, 1, 2, 3 and 4 in 30.7%, 35%, 16.4%, 14.3% and 3.6% of cases, respectively. Infrared-reflectance SLO images (IR) obtained the score of 0, 1, 2, 3 and 4 in 0.7%, 20%, 65.7% 9.3% and 4.3% of cases, respectively. Blue-reflectance SLO images (BR) obtained the score of 0, 1, 2, 3 and 4 in 7.1%, 5.7%, 25%, 44.3% and 17.9% of cases, respectively. En Face OCT images (En Face) obtained the score of 0, 1, 2, 3 and 4 in 2.1%, 0%, 29.5%, 52.9% and 15.7% of cases, respectively. *BR* blue-reflectance SLO, *En Face* En Face optical coherence tomography image, *CFP* color fundus photography, *IR* infrared-reflectance SLO

Regarding the preoperative images of all patients, the best score was obtained by En Face OCT (total score of 392) followed by BR images (total score of 364) and IR images (total score of 272). CFP obtained the worse score (total score of 171) (p < 0.001, Friedman test). Figure 2 shows the distribution of the scores for each imaging modality. The ERM limits or edges (score of 3 or 4) were visualized in 68.5% of cases on En Face OCT, in 62.1% of cases on BR images compared to only 13.6% of cases on IR images and 17.9% of cases on CFP. The ERM could not be identified in 22 out of the 70 cases (31%) on CFP because the image was not contrasted enough to distinguish either the ERM reflectivity or vessel constriction. ERM was not visible on BR images in 7% of cases (5/70), due to the presence of an artifact in 2 cases, to low-quality images in 2 cases, and to a very flat ERM in 1 case. In 3 cases, The ERM was not detected by En Face OCT in 3 cases (4%) because the ERM was thin and extended beyond the image dimensions.

The overall distribution of the scores for En Face OCT and BR images significantly differed according to the lens status (p = 0.0162 and p = 0.0094, respectively). The ERM limits and edges (grade 3 and 4) were more often visualized on En face images than on BR images, both in phakic and pseudophakic eyes (68.4 versus 63.3%, p < 0.001 and 69% versus 59.5%, p = 0.004, respectively). Regarding BR images, the ERM limits and edges (score of 3 and 4) were visualized in 63.3% of phakic eyes and in 59.2% of pseudophakic eyes, whereas on En Face OCT images, they were visualized in 69% of pseudophakic eyes and in 68.4% of phakic eyes. These differences were not significant (p = 0.6758 and p = 0.9367, respectively).

### Intraoperative image analysis

As the recording of the surgery was not systematically performed, an IDI was available in 26 out of the 70 eyes with a complete pre-operative imaging. Six eyes were excluded because they underwent phacovitrectomy to avoid the bias of an improved visualization after lens extraction for the comparison with preoperative images. In this subgroup of 20 eyes, patient mean age was 69.9 ± 9.1 years (range: 53–87 years) and 50% of patients were phakic. Patient characteristics are shown in Table 2.

**Table 2:** Pre- and intraoperative characteristics of the 20 patients with idiopathic epiretinal membrane with an intraoperative digital image available

Number of eyes, n	20
Age (years)	69.9 ± 9.1 (53–87)
Male gender, n (%)	6 (30)
Side: Right eye, n (%)	12 (60)
Preoperative BCVA, LogMAR Snellen equivalent	0.45 ± 0.24 (0.2–1.0) 20/63
Lens status, n (%)	
Phakic	10 (50)
Preoperative parameters on the OCT B-scan	
CMT (µm)	469 ± 111 (267–708)
Retinal folds, n (%)	14 (70)
ERM edge, n (%)	8 (40)
Pseudo-hole, n (%)	1 (5)
Complete PVD, n (%)	15 (75)
ILM peeling, n (%)	16 (80)

Quantitative values are presented as a mean ± standard deviation (range)

*BCVA* best-corrected visual acuity, *CMT* central macular thickness, *ERM* epiretinal membrane, *ILM* internal limiting membrane, *PVD* posterior vitreous detachment

**Fig. 3:**
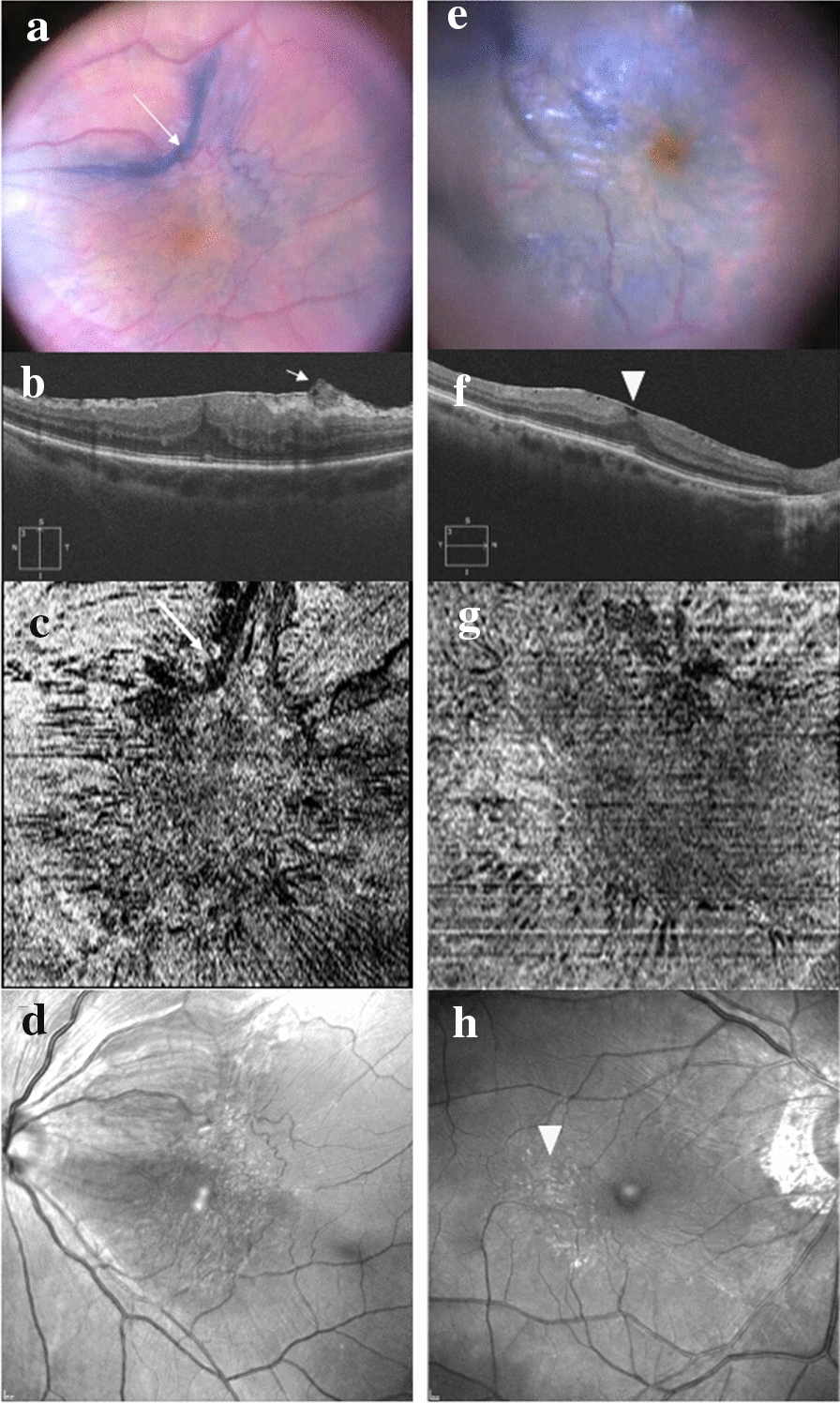
Different preoperative morphological aspects in 2 cases of idiopathic epiretinal membrane (ERM) using multimodal imaging. In case 1 (**a**–**d**), the arciform blue edge of the ERM (arrow) is clearly visible intraoperatively after staining (**a**), and corresponds to the edge identified on preoperative OCT B-scan (**b**) and En Face OCT (**c**) images (arrow) where it limits the plaque-like aspect of the ERM. The BR image (**d**) shows the ERM limits but the edges are less visible. In case 2 (**e**–**h**), the limits of the ERM are not clearly identified on the intraoperative image after staining (**e**). En Face OCT (**g**) and BR (**h**) images show a uniform retinal folding and a discrete reflectivity of the ERM (arrowhead) and provide a more accurate visualization of the ERM limits compared to the uniformly blue intraoperative staining (**e**). The OCT B-scan image (**f**) shows a thin ERM (arrow)

The ERM visualized on the IDI were divided into 3 patterns according to their aspect after a 1-min staining with Membrane blue Dual®, as follows: (1) uniform, defined as a homogeneous faint blue staining of the whole macular surface (Fig. 3e); (2) negative, defined as an intense blue staining of the internal limiting membrane (ILM) surrounding the reflective plaque of the ERM (Fig. 1b); and (3) blue edge defined as a blue arciform staining of one edge of the ERM (Fig. 3a). The intraoperative aspect of the ERM was considered uniform in 9/20 eyes (45%), negative in 6/20 eyes (30%) and blue edge in 5/20 eyes (25%). The latter 5 cases also all showed a negative aspect on the IDI.

The comparison of pre- and intraoperative images showed that En face OCT images was similar with the IDI image in 90% of eyes (n = 18/20). In detail, En Face OCT showed a diffuse retinal folding in 8 of the 9 eyes (89%) with uniform ERM staining. In these eyes, the OCT B-scan showed a widely spread hyperreflective structure without retinal folds or visible edges in 5/9 eyes (56%) (Fig. 3e–h). Similarly, En face OCT showed a plaque-like aspect in 10 of the 11 eyes (91%) with negative ERM staining (Fig. 1c). Also, the analysis of the OCT B-scans showed that the plaques corresponded to an area of attachment between the ERM and the ILM in 9/11 cases (81%). Regarding the ERM edges, when a blue edge was visible on the IDI, it corresponded to a detached or scrolled edge visible on the OCT B-scan in all cases (5/5) (Fig. 3a–d). However, when a detached or scrolled ERM edge was seen on the OCT B-scan (40%; 8/20), it was stained on the IDI and visible as a blue edge in only 5/8 cases (62.5%).

## Discussion

In a series of 70 eyes with idiopathic ERM, we scored all preoperative images in order to determine the best image to be used for visualizing ERM limits and edges. We found that the ERM morphological features were better visualized on En Face OCT and BR SLO images. In a subgroup of 20 patients, the comparison of preoperative En Face OCT and BR SLO images with the IDI acquired after staining showed similar aspects of the ERM but the blue staining did not always show the ERM edge.

En Face OCT has already shown its usefulness in characterizing ERM morphology as multiple small plaques in macular pseudo-holes, or its centrifugal or centripetal aspects [4, 6], and anatomical quantitative features such as the area of the plaque may be used as functional predictive factors [7]. In our series, the En Face OCT images obtained the highest score and allowed performing the best analysis of the ERM morphology in all cases. The BR SLO images also allowed a good visualization of ERM morphological features, especially in phakic eyes, in which a high score was more frequently obtained than on En Face OCT images. While using different wavelengths and techniques, both En face OCT and BR SLO allow visualizing the reflectivity of retinal or epiretinal structures such as ERMs. Nevertheless, the resolution of En Face OCT is lower than that of BR SLO, explaining why BR was more helpful in some cases. The BR SLO image combines the contribution of the confocal SLO technique and the BR wavelength (486 nm) which enhances the reflectivity of the inner retinal layer. Green reflectance and BR have been shown to be superior to IR and autofluorescence for identifying ERM edges and retinal folding surface [5, 8, 9]. The BR image could be a good candidate to be integrated as a filter in operating microscopes or as a display in the intraoperative visualization system to identify ERM limits without staining. Nevertheless, in our series, the score of En Face OCT images was slightly superior to that of BR images, probably due to a poorer quality of BR images that could be affected by a central reflective artifact of the intraocular lens (IOL) in pseudophakic eyes. Based on the BR SLO wavelength, the presence of an IOL filtering wavelength less than 500 nm could potentially explain this artifact.

Also, BR and En Face OCT images were superior to CFP and IR for characterizing ERM morphology in all cases in line with recent studies [10]. In 31% of cases, CFP failed to identify the ERM as previously reported [5]. Therefore, CFP does not seem to be the most useful preoperative imaging technique for routine ERM analysis, whereas En Face OCT and BR SLO images should be routinely integrated in ERM preoperative imaging.

The intraoperative method currently used to identify ERM limits is based on the use of vital dyes such as blue dyes or indocyanine green, depending on the approval of these different dyes by the local authorities. In our French series, only blue dyes have obtained the CE marking (trypan blue and brilliant blue G). The qualitative analysis of our subgroup of 20 patients with IDI available showed that after staining, the IDI was not always the best image to be used for visualizing the ERM. In 45% of cases, the blue staining was faint and uniform in the macular area, preventing the surgeon from clearly identifying ERM edges. Also, the detached or scrolled edges visible on the OCT B-scan images were not always visualized in blue after staining. In our series, the IDI after staining was not consistent with the preoperative en face OCT image in 10% of cases. We observed that the staining aspect depended on the ERM morphology characterized preoperatively: when the blue dye stained uniformly the ERM, the En Face OCT image showed a diffuse retinal folding in most (89%) cases. Wide plaques visible on En Face OCT corresponded in most cases to a negative aspect of the ERM staining seen intraoperatively (91% of cases). In fact, brilliant blue G only stains the outer surface of the ILM which takes an intense blue color. This could explain the blue staining of some detached or scrolled edges. But brilliant blue G has a low affinity for the ERM that is slightly stained with trypan blue, giving this patchy negative aspect [2, 11]. This plaque-like aspect was found in 55% of cases and corresponded to areas of adherence with the ILM, as reported by Rispoli et al. [4]. These areas were therefore not suitable for initiating ERM peeling [4].

The challenge of vitreoretinal surgeons in future ERM surgery will be to perform a more precise peeling, allowing preserving the ILM and possibly avoiding the use of dyes. Indeed, it has been shown that removing the ERM in eyes with good preoperative visual acuity allows a better visual rehabilitation [12, 13]. The fact that the ILM is more intensely stained could encourage surgeons to initiate ERM peeling in these areas, when the ERM edge is located at the limits of the stained area. Thus, it frequently leads to ILM peeling as seen in 80% of our cases. On the one hand, some surgeons recommend systematically performing ILM peeling to prevent ERM recurrence [14]. Except a different recurrence rate, no study has reported a significant difference in BCVA or central macular thickness when the ILM was peeled or not [15]. On the other hand, a concern has arisen regarding the effect of ILM peeling on visual acuity, contrast sensitivity and the appearance of microscotomas [16]. Sometimes, regardless of the surgeon’s intention, the ILM peels off simultaneously with the ERM. Seidel et al. have reported that a greater ERM elevation with a looser connection between the ERM and the retinal surface and thicker ERMs on SD-OCT were predictive of ILM persistence after ERM peeling [17]. The analysis of the retinal adherence to the ERM and the identification of a detached or scrolled edge on preoperative images could allow surgeons to identify the best area to initiate ERM peeling and therefore to avoid retinal trauma [18].

Recent innovations in intraoperative visualization are intended to provide multimodal retinal imaging information during surgery. An intraoperative OCT platform provides OCT B-scan images before, during and after ERM peeling, helping surgeons to identify areas where ERM peeling may be initiated with the lowest risk of retinal contact, and to confirm the complete removal of the ERM and/or ILM without using a second dye [19]. The new heads-up 3D visualization system, consisting in an image acquisition with a 3D high dynamic range surgical camera and the display on a 46″ high-definition LCD screen [20], offers many filter options to enhance epiretinal structure contrast and also allows concurrently displaying preoperative images [21, 22]. Therefore, the information provided by preoperative En Face OCT, BR or OCT B-scan images, displayed on the intraoperative live image, could guide surgeons and maybe reduce the need for dyes [22].

In this study, we compared preoperative images of the ERM, the quality of which depended on the OCT device resolution. That is why we chose the best technology available to acquire all images, from the use of the latest versions of reference OCT devices to the intraoperative visualization using high-precision operating microscopes, in order to obtain the best quality images for analysis. Also, when the ERM can be visualized in 3D on fundus examination and during surgery, reducing it to a 2D analysis on a digital image is associated with a loss of information. Regarding the limits of the comparison of preoperative images with the IDI, as images were taken at different times, we cannot exclude an evolution of ERM morphology over the time interval between the preoperative imaging and the surgical procedure. ERM is a common disease for which routine imaging protocol is not as complete as in this study and intraoperative live is not systematically captured. With its selective inclusion criteria and the retrospective design of the study, the population for which intraoperative and preoperative images could be compared may appears small. However, the concordance between the morphology of the ERM on preoperative and intraoperative was of 90%.

In summary, ERM morphology was better seen preoperatively on En Face OCT and BR SLO images and in most cases, these images were highly similar to the intraoperative image after blue staining. Remarkably, in some cases, especially when the ERM was wide and spread across the retinal surface, the IDI after blue staining did not allow a proper visualization of ERM limits while they were visible on other imaging modalities. An optimized visualization of the ERM limits and edges on preoperative images is crucial for a good planification of the surgical procedure. In future intraoperative visualization systems, displaying these preoperative images during surgery could enhance the surgical precision, help to initiate ERM peeling and avoid the use of dyes.

## Data Availability

The datasets used and/or analysed during the current study are available from the corresponding author on reasonable request.

## Abbreviations

ERM: Epiretinal membrane
BR: Blue-reflectance SLO
En Face: En Face optical coherence tomography image
CFP: Color fundus photography
IR: Infrared-reflectance SLO
